# Recent Trends in Assessment of Cellulose Derivatives in Designing Novel and Nanoparticulate-Based Drug Delivery Systems for Improvement of Oral Health

**DOI:** 10.3390/polym14010092

**Published:** 2021-12-27

**Authors:** Khaled M. Hosny, Hala M. Alkhalidi, Waleed S. Alharbi, Shadab Md, Amal M. Sindi, Sarah A. Ali, Rana B. Bakhaidar, Alshaimaa M. Almehmady, Eman Alfayez, Mallesh Kurakula

**Affiliations:** 1 Department of Pharmaceutics, Faculty of Pharmacy, King Abdulaziz University, Jeddah 21589, Saudi Arabia; wsmalharbi@kau.edu.sa (W.S.A.); shaque@kau.edu.sa (S.M.); rbakhaidar@kau.edu.sa (R.B.B.); amnalmehmady@kau.edu.sa (A.M.A.); 2 Department of Clinical Pharmacy, Faculty of Pharmacy, King Abdulaziz University, Jeddah 21589, Saudi Arabia; halkhaldi@kau.edu.sa; 3 Department of Oral Diagnostic Sciences, Faculty of Dentistry, King Abdulaziz University, Jeddah 21589, Saudi Arabia; amsindi@kau.edu.sa (A.M.S.); samali@kau.edu.sa (S.A.A.); 4 Department of Oral Biology, Faculty of Dentistry, King Abdulaziz University, Jeddah 21589, Saudi Arabia; ealfayez@kau.edu.sa; 5 Department of Biomedical Engineering, The Herff Collage of Engineering, Memphis, TN 38152, USA; mallesh_kurakula@yahoo.com

**Keywords:** biopolymers, cellulose derivatives, excipient, nanoparticles, drug delivery, controlled release

## Abstract

Natural polymers are revolutionizing current pharmaceutical dosage forms design as excipient and gained huge importance because of significant influence in formulation development and drug delivery. Oral health refers to the health of the teeth, gums, and the entire oral-facial system that allows us to smile, speak, and chew. Since years, biopolymers stand out due to their biocompatibility, biodegradability, low toxicity, and stability. Polysaccharides such as cellulose and their derivatives possess properties like novel mechanical robustness and hydrophilicity that can be easily fabricated into controlled-release dosage forms. Cellulose attracts the dosage design attention because of constant drug release rate from the precursor nanoparticles. This review discusses the origin, extraction, preparation of cellulose derivatives and their use in formulation development of nanoparticles having multidisciplinary applications as pharmaceutical excipient and in drug delivery, as bacterial and plant cellulose have great potential for application in the biomedical area, including dentistry, protein and peptide delivery, colorectal cancer treatment, and in 3D printable dosage forms.

## 1. Introduction

Cellulose (C6H10O) is a polysaccharide; a carbohydrate polymer contains hundreds to millions of individual sugar molecules, or the so-called anhydrous-D-glucopyranose units (AGU) linked together via glucosidic linkages. It is a major component present in the plant cell wall, and often used as fiber in human diet [1,2]. Cellulose is widely occurring biopolymer of a natural origin. Cotton and other higher plants have natural fibers made up of cellulose as major constituent; it is well-established that its pure form is present in cotton seed at 90% weight. It may also be obtained from food waste and wood [2]. Cellulose as polymer contains as chains [3]. Of note, the first synthetic polymers were composed of cellulose, including cellulose nitrate, cellulose citrate and rayon. Individual cellulose moiety has three to four hydroxyl –OH-groups according to the AGU, except for last molecule. Lately, it proved to possess versatile applications in paper industries, textile as well as in the pharmaceutical field. Cellulose, as an excipient, complies with food and drug administration (FDA) act requirements of pharmaceutical or chemical excipients such as enhanced stability, being eco-friendly resource, easily renewable and non-toxic for the purpose of development of conventional dosage forms [3]. Cellulose and its components are partially or water insoluble and even in most organic solvents; this poor solubility is primarily due to the stronger intra-molecular and inter-molecular hydrogen bonds that are present between the branched or individual chains [4], however, cellulose derivatives are soluble in cold water (Wilpiszewska et al., 2020). It is characterized by being odorless and tasteless (Zainal et al., 2020). Cellulose and their derivative are broadly used for building construction; manufacturing of paper and their productsas well aswood material, extraction and preparation of rayon, cotton and linen for fabricating clothes, chemical extraction of nitrocellulose for developing explosives; cellulose acetate clarification for manufacturing films and many other products. Additionally, they have further versatile applications in biomaterials, electronics and electrochemical devices (Pandi et al.).

## 2. Cellulose and Their Derivatives

**Figure 1:**
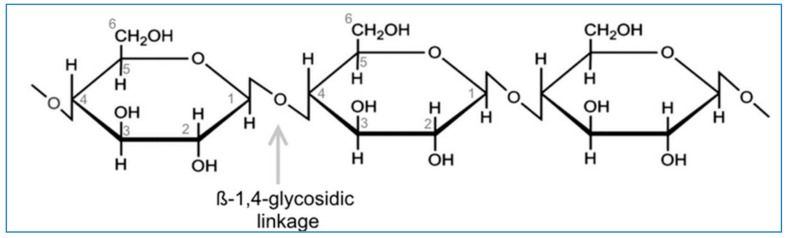
Schematic representation of β 1,4 glycoside linkage in glucose units.

Cellulose abundantly exists within the wall linings of cells of bacteria such as Cyanobacteria species, few prokaryotes such as Acetobacter, Rhizobium, Agrobacterium species, and even in eukaryotes like fungi, amoebae, gymnosperms genre. Cellulose components are assimilated or generated by few tunicates such as urochordates, even by few organisms belonging to the subphylum of Tunicates especially the Chordata phylum [5,6,7]. The biosynthesised cellulose is major of glucose units, like glucose units (C_6_H_10_O_5_,n) or AGU consisting of uniform organised β-1,4 linkages (Figure 1).

**Figure 2:**
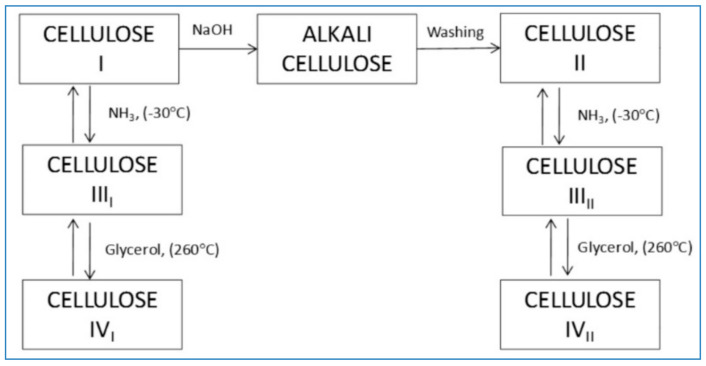
Schematic representation of preparation of different allomorphic forms of cellulose.

Forms of Cellulose and its derivatives: Cellulose existence has both cellulose I (greenly plants) and cellulose II (lower phyla or bacterial or algal species) as allomorphs the orientation of the AGU units are either antiparallel or parallel respectively [8,9]. Allomorphic forms of cellulose are I, II, IIII, IIIII, IVI, IVII. These forms exhibit difference in structures such as cell dimensions, intra/inter hydrogen bonding among cells and cellulose adjacent polarity [10]. Cellulose I resemble nanofibrils since the glucose units are parallel arranged. (Figure 2) Indicated that natural cellulose in crystalline form I has two sub allomorphs, α and β, that are single chain and in triclinic or monoclinic unit arrangement.

Cellulose I β is thermodynamically stable and is rarely synthesized by tunicates [11]. With alkali treatment Cellulose I can be modified or converted into crystalline structures such as II, III and IV. Cellulose II existence is highly stable among I, III and IV [12,13,14]. Cellulose III is prepared using ammonia (liquid) or when cellulose I or II forms is amine treated [15]. Cellulose IV exists in crystalline morphology and is formed when cellulose III is immersed and heated in glycerol [16]. Cellulose is commercially available in powdered (n500) and microcrystalline (MCC) (n220) forms. Microcrystalline (MCC) is obtained when cellulose nanofibrils are acid hydrolysed. The formation of various cellulosics, like methyl, ethyl, hydroxyethyl, hydroxyethylmethyl, hydroxypropyl (HP), hydroxypropyl methyl (HPM/hypromellose) and carboxymethyl ethers is when the wood pulp is hydroxyl etherified in the presence of alkyl halide (R-Cl) [17]. The degree of substitution (DS) within ether derivatives resembles the glucan and count of R group’s as substituents. DS has major influence on physical properties like solubility [18]. When acetic, trimellitic, dicarboxylic phthalic or succinic acids are hydroxyl esterified several cellulosics, such as acetate, acetate trimellitate, acetate phthalate (CAP), HPM phthalate, HPM acetate succinate are by produced, generally all these reactions are performed in the presence of a strong acid. Among these cellulosics, CAP recognised for pH-controlled release [19]. These cellulosics are gastroretentive and useful in enteric coating for capsules or tablets [20,21].

Chemical modification of cellulose has led to the production of nanocrystal cellulose (NCC) cellulose nanofibers (CNF) and a number of othe cellulose thermoplastics [2].

## 3. Origin

Carboxymethyl cellulose (CMC) is generally isolated, extracted and recovered from palm kernel cake (PKC) and modified into CMC. Following the extraction of oil palm, oil palm fronds (OPF) also has been also recycled. Of note, OPF is composed of 58% cellulose, 24% hemicellulose, 5% lignin, 8% extractive and 5% ashes. Fortunately, OPF may possibly be used as cellulose materials and be an alternative material for composites (Zainal et al., 2020). Di or tri acetate cellulosic polymers, are obtained by purification from various bioagricultural wastes such as oil palm branch and fibers after the oil extraction, piassava, pulp of bamboo, bark from raphia bamboo palm, stems and cob of maizes, fibers of fruit like coconut, sawdusts extracted from sugarcane or pear wood or cotton and few stems from plantain [22]. Numerous methods have been used in order to extract cellulose from bio-waste, for instance steam explosion, Organo-solv method, ionic liquids method, and chlorine-free method. Nonetheless, each method has its downfalls that make the process of high cost with the production of less cellulose. Cellulose acetate and CMC are extracted from sugarcane straw; the microcrystalline cellulose (MCC) obtained within the raw cotton of ochlospermum planchonii; microfibrillated celluloses (MFCs), obtained from different yields of wood pulps; nanofibrillated cellulose (NFC), is extracted from pulp of wood yet manufactured by making water dispersions of NFC. These dispersions needto be solvent exchanged from aqueous water to tert-butanol and even by lyophilization; bacterial cellulose, from bacterial pellicle [23].

## 4. Extraction Procedures

### Liquid Phase Oxidation for Extraction of Cellulose from Palm Kernel Cake (PKC)

**Figure 3:**
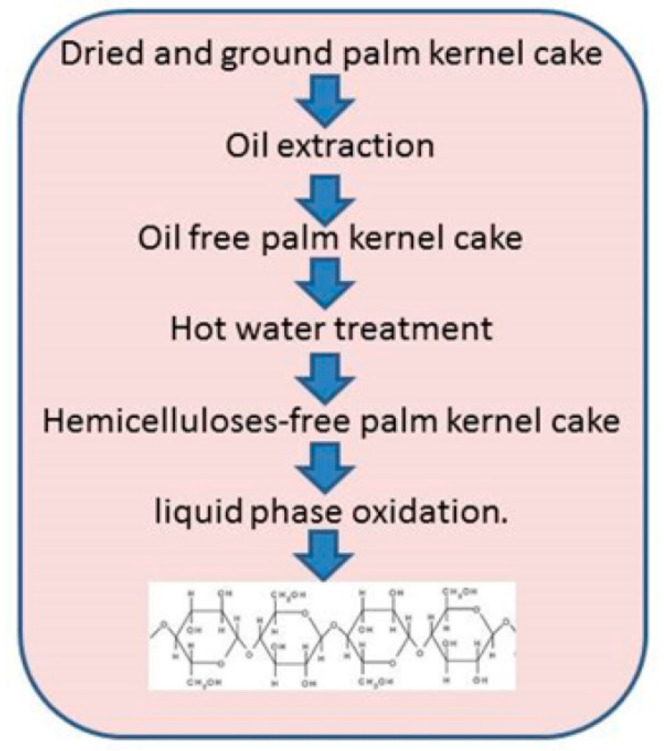
Scheme representing extraction of cellulose from palm kernel cake.

In cellulose extraction, using hot water treatment is pretreatment step. PKC of approximately 6 g weight is grinded with 30 mL of distilled water and taken in a micro-pressure reactor. The temperature is maintained at 160–180 °C, subsequently there is an increase in reaction pressure up to saturated vapor pressure. After an hour with the cool water the reactor is soaked in order to stop the reaction. The pretreated PKC is washed thoroughly using water and next the ethanol is used to remove the remaining organic acids and other saccharides. Further, the mass is then dried and subsequently sieved to 600 mm. Lignin is removed by using liquid phase oxidation from PKC in order to obtain pure cellulose. Approximately, 2 g of PKC pretreated is thoroughly mixed with 30% hydrogen peroxide (H_2_O_2_) in the ratio of 1:5, 1:7.5, and 1:10 (weight basis) tightly plugged within conical flask. The mixture reaction is maintained from 60–80 °C for about 10–24 h. After the time elapses, cold water in excess will be added in order to stop the oxidation reaction in the mixture (Figure 3). Finally, the mixture is filtered and the solid residue is isolated from the organic compound solution [24].

## 5. Extraction from Agricultural Waste

Cellulose is extracted from dhaincha dried barks, stalks of corn, from straw of rice and wheat. Formic acid treatment conditions are formic acid concentration 90% (*v*/*v*) reaching boiling point for 120 min. The fibers are washed with fresh formic acid and treatment with peroxyformic acid is done by attrition of 90% formic acid and 4% H_2_O_2_ and pH is adjusted to 11, by adding (NaO) and further hydrolyzed using 63% *w*/*w* conc. Sulphuric acid solution for 6 h at 45 °C, with constant stirring. Washing of the final mixture with cold water is performed; centrifuged and then residue is vaccum dried about 47 to 48 h [25].

## 6. Extraction from Wood

Approximately 100 mg of dry plant tissue (finely grounded) is taken into a 2-mL Eppendorf tube. Then 1 mL of diethylene glycol dimethyl ether (diglyme) and 0.25 mL 10 M HCl is added. The eppendorf tube is further incubated at 90 °C in a water bath for 1 h. Even distribution of samples is required during incubation. Further, cool the samples, then centrifuge and discard the fine supernatant. Each pellet is washed three times thoroughly with 1 mL methanol and 1 mL hot distilled water. The residues are dried at 80 °C and weighed into capsules (tin) [26].

## 7. Extraction from Cotton

Pure cotton is weighed and transferred into a round bottom flask, fitted with water condenser. 1 N NaOH is added to round bottom flask and heated at 105 °C for 1 h. It is subjected to bleaching using H_2_O_2_ and then hydrolyzed using 2.5 N HCL. Slurry is cooled and filtered and residue is dried in an oven at 40 °C overnight [27].

## 8. Extraction from Bacterial Pellicle

Bacterial pellicle is thoroughly washed with water and cut in to small pieces and transferred into a round bottom flask and then added with 1N NAOH and heated at 105 °C. Product is bleached with H_2_O_2_ and then hydrolyzed using 2.5 N HCL. This mixture is washed with distilled water, filtered and the residue is dried in an oven at 40 °C overnight [28].

Preparation of surface-functionalized cellulose nanocrystals by cellulose esterification followed by hydrolysis using solvent-free reaction (using oxalic acid dihydrate):

The first step is performed by milling followed by dissolving softwood oxalic acid pulp dihydrate (molten) at 109 °C about 15, 30, 60, and 120 min, with uniform mixing and under stronger agitation reflux, to isolate cellulose oxalates (COX), commonly named as COX15, COX30, COX60, COX120, respectively. Here, oxalic dihydrate is used as an esterification agent for wood fibers, whereas its dehydrating product is applied in different solutions in the hydrolysis of cellulose in the cellulose nanocrystals preparation. Here, oxalic acid dihydrate is used because of its relatively lower melting point of 105–107 °C, therefore possible to perform a solvent-less reaction. The molten oxalic acid dihydrate wets the fibers, resulting in combined hydrolysis and derivatization of cellulose. All samples should be filtered, washed and extraction using Soxhlet was performed in order to filter the ruminants of oxalic acid dihydrate. Cellulose oxalates are fine white powder obtained after drying [29].

## 9. Physiochemical Properties of Different Cellulose Derivatives

Table 1 shows examples of cellulose derivatives and their chemical structure, molecular weight, viscosity, solubility, and uses.

## 10. Recent Trends in Cellulose Derivatives-Based Nanoparticule Drug Delivery Systems

Nanotechnology provides a unique solution to obligations faced by the conventional dosage forms; this offers a booster alternative for superior patient health and quality of life [36]. Nanoparticles (NPs) intended for use as drug carriers are of different types, of which solid lipid NPs (SLNPs), dendrimers, nanocapsules and nanospheres are essential forms, other types include inorganic carbon-based gold or iron oxide and silicon-based NPs, and fullerene (Bhatia, 2016) They are prepared from different material types; amongst these, b natural synthetic and semi-synthetic based polymers are extensively used in pharmaceutical sector. Natural polymers include albumin, alginate, or chitosan have been widely researched (Zazo et al., 2016), while synthetic polymers include polylactide, polylactide–polyglycolide copolymers, polycaprolactones, and polyacrylates (Daniel, 2021). Noteworthy, the selection of such NPs for use as drug vehicles should meet the FDA criteria for human use. Predominantly, the above-mentioned polymers should be biodegradable and biocompatible in order to be eliminated from the body in a short duration and no risk of accumulation. Also, the polymers and their by-products obtained from metabolism, should not be toxic or cause any auto immune response [37].

Cellulose-based NPs have gained prominence in formulation development due to the various beneficial properties proven to be offered by them such as:

- Increased drug solubility and stability, thus prolonging systemic circulation for an enhanced bioavailability.
- Site-specific targeting to the pathogenic tissues and organs.
- Aids in sense of sensitivity to the environmental stimuli (alterations in pH, heat, magnetic field or ultrasound), or to the invasive pathogen stimulation (alterations in temperature, redox environment)
- Transferring the vital transporters that can provide metabolism information even to target an organ system [38,39].

Suk Fun Chin et al. (2018), used nanoprecipitation technique to prepare methylene blue-loaded spherical cellulose NPs of 70–365 nm average sizes. The study demonstrated that both cellulose concentration and volume ratio of non-solvent to solvent are the major factors influencing the diameter of the NPs. Additionally, it was found that the NPs’ diameter affects both the drug release profile as well as the loading efficiency [40].

Akbar Esmaeili et al. (2017), conducted comparison tests of modern medicines along with two herbal based drugs using nanoparticles made of starch-combined cellulose shell having drug loaded in alginate space. The optimal specifications for precursors of these nanoparticles are reported as 0.01 g cellulose, 0.15 g starch along with alginate approx. 0.04 g, at pH 4, those are spherical in the range of 25.6–68.9 nm. The reported technique offers a big advancement in bone regeneration in osteoporosis treatment. The drug release was profoundly sustained [41].

Yi-HsuanTsai (2018) experimented on bacterial cellulose, a biopolymer that contains excellent film-forming ability due to the internal composition of nanofibers. The applicability this cellulose is for the food and biomedical applications only as it doesn’t show any antibacterial or antioxidant activity. The study reported that silymarin (flavonoid) along with zein can assemble into nanoparticles (spherical) so that flavonoid can get adsorbs over cellulose nanofibers. The swelling nature and extent of wettability of bacterial cellulose based film membranes changed greatly because of the formation of silymarin -Zein nanoparticles composed nanofibers as nanocomposites [42].

According to a work by Sonal Mazumder, polysaccharide-based NPs of 150–200 nm average size were formulated using a rapid precipitation process for poorly soluble antiviral drugs; the enhancement of solubility of the aforementioned drugs was studied by structure-activity relationship (SAR) for 4 newly synthesized cellulose-acetate polymers. Drug loading into the particles was expected to be 25% of weight, while the calculated drug loading was observed as 80–96%. Compared to the pure drugs, these NPs demonstrated improved solubility and faster release. The study revealed that release rate of the drug is a function of the relative hydrophobicity of the used polymers [43].

Ahmed salama (2015) recognized the immediate need to subside the bacterial infections resistant to antibiotics. Silver NPs (15 nm) decorated with 2, 3 dicarboxylic cellulose were reported. The decorated Dicarboxylic cellulose-based silver nanocomposite proved enhanced antibacterial action against both gram positive/negative bacteria [44].

In a recent work, researchers have prepared controlled release drug delivery systems by using a newer bionanocomposite that are pH-sensitive beads prepared with sodium carboxymethyl cellulose (Na-CMC) and Zinc oxide nanoparticles (ZnO NPs). UV–vis spectroscopy was used to determine propranolol loading efficiency within the beads and was found to be high. The bionanocomposite beads showed greater water absorbing capacity between pH 7.2 to 7.4. The ratio of swelling for ZnO-based CMC hydrogels performance in different water solutions was greater with respect to plain hydrogel. Drug release in-vitro indicated controlled and even release similar to that were observed for zinc oxide nanoparticles having Na-CMC based bead that increased with change in zinc oxide nanoparticles concentration [45], ZhilaZare-Akbari (2016).

Moreover, YongboSong (2015) prepared nanoparticles using CMC and quaternized cellulose (QC) having different charges. The surface charge influence over cellulose NPs and their ability to deliver either positive or negative charged biomolecules as proteins was studied. Bovine serum albumin (BSA) and Lysozyme (LYS) having positive (+ve) and negative charge (−ve) were used as models at physiological pH respectively. Their work has demonstrated reported that efficiency of loading achieved was high about 67.7% and 85.1% even when a negative charge protein has been encapsulated within a NP of opposite charge, or vice-versa. Low cytotoxicity and high cellular uptake efficiency of charged NPs were exhibited due to good biocompatibility of cellulose [46].

## 11. Bacterial Cellulose

Cellulose is biosynthesized by various species of microorganisms called as bacterial or microbial cellulose and plants [47]. Several species of bacteria including Pseudomonas putida (P. putida), Erwinia chrysanthemi, Escheria Coli (E. coli), Dickey dadantii and Burkhoderia spp. In addition to these microorganisms several other species of the bacteria belonging to the genera of agrobacterium sarcina and rhizobium are extensively evaluated for the production of cellulose [48]. A study conducted by Klemn et al., 2001 and Ullah et al., 2016 reported that gluconacetobacter, a gram negative aerobic bacteria has the ability to produce cellulose in huge quantity as compared to the other species of bacteria [49,50].

**Figure 4:**
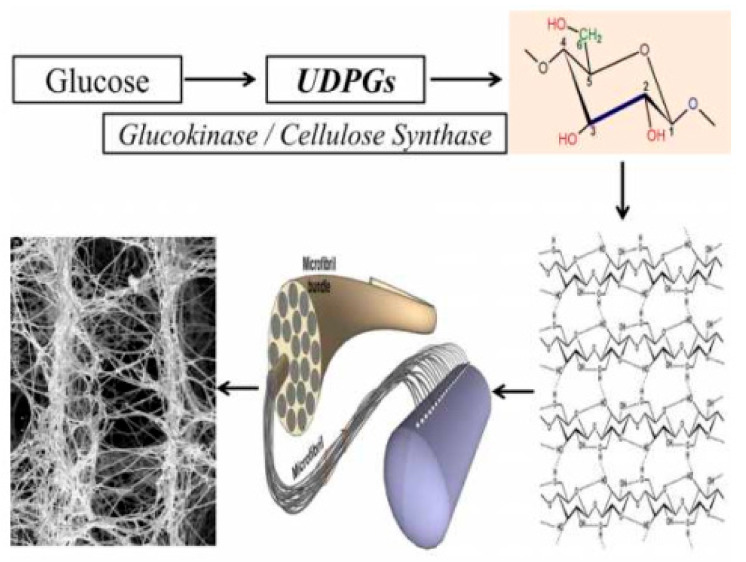
Structural organization of bacterial cellulose. A general overview (Courtesy Suleva et al., 2015). Characterization of bacterial cellulose (BC) or regenerated BC (RBC) based drug delivery.

Figure 4 shows the Structural organization of bacterial cellulose, microorganism utilize different carbon sources like glycerol, dicarboxylic acid, hexoses and pyruvate for producing cellulose as exo-polysaccharide. The process of cellulose production by bacterial species is complex that involves several enzymes and proteins. The mechanism for the synthesis of uridine di phosphate glucose is well investigated by Ebrahim et al. in 2016 [51]. According to their study, the sugar molecule is converted into uridine di phosphate glucose and the precursor is excreted in the form of cellulose using enzyme cellulose synthase. This mechanism leads to produce subfibrils followed by the micro fibrils which then adopt the shape of hundreds to thousands ribbons. Mechanism and process for the production of bacterial cellulose have been explained in the below Figure 4 [6]. Table 2 depicts the various parameters/characterization which are carried out for BC or RBC based drug delivery system [52].

## 12. Role of Cellulose Derivative in Formulation of Novel Drug Delivery Systems

Liu, Mengqi, et al. (2017), developed a pH gradient releasing pellet of Vinpocetine (VIN) extrusion spheronization technique with self-emulsifying drug delivery system (SEDDS). Three coating materials such as HPMC, Eudragit L30D55 and Eudragit FS30D have been used in the study. The dosage form improved the oral bioavailability by approximately 149.8% in comparison to commercial VIN tablets and to reduce the plasma concentration fluctuation. The research has indicated that absorption of VIN is pH-dependent [53].

Rao, Ziqie, et al. (2018), have incorporate biological amino-based molecules within graphene oxide (GO) to group a complex as amino-fumed graphene and to combine this complex with CMC in order to generate graphene-CMC complex that acts as a drug embossed carrier matrix. Doxorubicin (DOX) was loaded within graphene -CMC forming graphene-CMC/DOX system. At pH 5, the drug release was about 65.2%. A cytotoxicity study on cervical cancerous cells (human Hela cells) and fibroblasts of mouse (NIH-3T3 cells) was negative [54].

McKenzie, Barbara, et al. (2015), conducted preformulation studies of cysteamine gels that can be useful in the treatment of cystinosis. Corneal crystal accumulation is the major complications which arised due to hourly administration of cysteamine eye drops in patients. Cysteamine gels were prepared and analyzed for their rheology, dissolution and stability properties. Sodium hyaluronate indicated good bioadhesion releasing for about 40 min and better stability and release properties in comparison to hydroxyethyl cellulose and carbomer 934 [55].

Firstly, Tania, et al. (2017), had formulated and characterized a solid support like sponges, that are produced by lyophilization of cellulosic derivative (HEC 250 M) hydrogels. The cellulose based sponges were characteristic in adhering around the vaginal cavity and were rehydrated by interaction of the vaginal fluids (small amount). The studies indicated that hydrogel viscosity and mucoadhesive strength were mainly dependent on the HEC concentration and were able to achieve a prolonged local drug effect [56].

Bezerra, Roosevelt DS, et al. (2016), developed a new phosphated cellulose (PC) for the amitriptyline release. The study explained the role of cellulose phosphate in entrapment and release of amitriptyline. Phosphated cellulose was prepared by the reacting cellulose (pure) with sodium (Na) trimetaphosphate (P) reflux at 393 K about 4 h. The drug adsorption capacity was increased up to 102.27% in cellulose with the help of phosphating. At pH 7, amitriptyline release was up to 40.98% ± 0.31% indicating pH dependence [57].

Tan, Huan, et al. (2015), used freezing-thawing method to prepare novel spongy collagen cryogels using natural cellulose derivative dialdehyde carboxymethyl cellulose (DCMC). The microscopy images indicated macropores as heterophase structure. The stability of collagen was found to be increased by incorporation of DCMC The swelling ratio was found to be pH dependent. The overall research performed using non-toxic materials such as DCMC that is cost effective with broader application in tissue engineering [58].

Priotti, Josefina, et al. (2017), prepared microcrystal formulations using chitosan, cellulose derivatives of Albendazole via bottom-up technology in order to enhance the solubility, dissolution rate thereby the antiparasitic activity. The in vitro evaluation of anthelmintic activity on adult forms of Trichinella spiralis identified system S10A was profound [59].

Tanaka, Akiko and co-workers (2016) have studied the influence of sodium carboxymethyl cellulose (Na-CMC) on absorption of fluorescein isothiocyanate-labeled dextran (Mw 4.2 kDa) nasally as well as insulin. Both solution form and starch powder form were examined. The absorption of powder possessing 50% starch (control) was greater than that following the application of the solution form and was even further increased by the starch substitution with Na-CMC. The insulin nasal absorption from the powder and the Na-CMC effect of were similar with FD4. With CMC-Na no dysfunction of the mucociliary clearance was observed, indicating an advanced strategy to enhance the macromolecule nasal absorption of [60].

Further, the same researchers have/Tanaka, Akiko, et al. (2017)/investigated the influence of three different types of HPC with various polymerization degrees such as HPC (SL), HPC (M), and HPC (H) on nasal drug absorption of powdered forms of warfarin (BCS Class I), piroxicam (BCS Class II), and sumatriptan (BCS Class III). The study demonstrated enhanced piroxicam absorption by HPC (M) while sumatriptan absorption was increased using both HPC (M) and HPC (H). The study has also revealed that absorption through nasal route is proportional to permeability and drug solubility, and that HPC derivatives can greatly influence the drug nasal absorption [61].

Liakos, Ioannis L., et al. (2016), utilized cellulose acetate to formulate nanocapsules using anti-solvent method in order to enhance antimicrobial properties of lemongrass oil. The formulated nanocapsules were 95 and 185 nm exhibiting good bioadherence and antimicrobial activity against Escherichia and Staphylococcus species [62].

Pastor, Marta, et al. (2014), formulated Oral cholera vaccine (OCV) gastro-resistant powder as an alternative to avoid the well-known transport and storage issues. Using cellulose acetate phthalate (Aquacoat^®^ CPD) as the core polymer, Attenuated Vibrio cholerae (VC) was entrapped using spray-drying technique. The microparticles (MPs) formed were in the range of 6 µm indicating 8.16 to 8.64% encapsulation. Two different strengths powders were prepared 30 and 60 mg, and a greater stability was exhibited at 80 °C in comparison to the corresponding suspension. The obtained results appeared to be quite promising towards formulation of powder-form Cholera vaccination (Pastor et al., 2014) [63].

Fekete, Tamás, et al. (2017), cross linked CMC using high-energy irradiation to prepare starch based superabsorbent hydrogels. Starch content in higher proportions resulted in negative impact on the rate of gelation, this actually greatly decreased the gel fraction. Hydrogels having 30% starch exhibited water uptake of ~350 g water/gel prepared from 15 *w*/*w*% solutions. Due to the CMC concentration actually the hydrogels have indicated a strong sensitivity towards the ionic strength within water. High water uptake was observed in greater electrolyte environment. CMC based starch hydrogels are alternative to pure cellulose derivative-based gels [64].

Popov, Todor A., et al. (2017), prepared HPMC-p-based micronized powder to assesses both safety and efficacy aspects of the powder product (patented) in terms of providing a natural membrane barrier against noxious or pollen allergens. Allergen provocation exams namely in three clinical trials were performed and the rest on placebo-controlled designs. Efficacy was even measured to treat the local symptoms. Acute experiments showed that when HPMC-p is applied intra-nasally gives local relief and further even enhances body immune system to reduce symptoms as well as long term therapy use. HPMC-p when prepared in nasal insufflation powder has proven not only as a mucosal barrier but also in effectively minimizing the symptoms of nasal and even potentiating the local relief effect of the medications used [65].

Arca, Hale cigdem, et al. (2018), developed solid dispersions (amorphous) of Rifampin using ω-carboxyalkanoates (cellulose) and compared the bioavailability, gastric pH stability with Rifampin (crystalline and negative charged) and carboxymethyl cellulose acetate butyrate solid dispersions (positive) controls. Cellulose ω-carboxyalkanoate solid dispersions proved successful in preventing acid-catalyzed degradation in stomach at acid pH, and released entire Rifampin at intestinal pH [66]. This cellulose derivative matrice has created high Rifampin oral bioavailability in the treatment of TB.

Rashid, Rehmana, et al. (2015), examined the effect and influence of the Tween 80 and HPC on the both physicochemical properties and even solid dispersions of ezetimibe, oral bioavailability that formulated by using solvent evaporation method. The studies indicated that with increase in HPC concentration to 9-fold, even the dissolution of drug and solubility were even increased but with further increase did not result in any significance. Solid dispersions were in amorphous form and indicated 1.6 to 1.9% increases in oral bioavailability in rats when administered. The studies revealed that HPC based ezetimibe solid dispersions were best suitable for oral administration for better bioavailability [67].

Qi, Xiaole, et al. (2015), used hydrophilic polymer (hydroxypropyl cellulose) HPC to prepared a floating tablet by compression method and by coating in combination of sodium bicarbonate acting as an effervescent substance. This system was evaluated controlled release of ofloxacin. Majorly, the effect of drug: HPC (weight) and the HPC viscosity on the drug release rate were examined. The average floating time was 30 s and lasted up to 11–12 h in pH 1.2 fluid SGF (simulated gastric fluid), in absence of pepsin, reflecting a zero-order drug release. Ofloxacin’s bioavailability (relative) after the administration was found to be 172.19% in New Zealand rabbits in comparison to TaiLiBiTuo^®^. Overall studies indicated that floating tablet coated with HPC as promising gastro-retentive delivery system [68].

Dual-drug delivery system based on the hydrogels of alginate and sodium carboxymethyl cellulose for colorectal cancer treatment.

Adenocarcinoma of the colon is considered highly prevalent nowadays which is the second most common cause of cancer related deaths worldwide [69]. It has been reported that 50,000 deaths alone in US and 20,000 deaths in Italy are caused by colorectal cancer. The death ratio due to colorectal cancer is more in male than female of age 50–60 years. Conventional chemotherapy based on oral drug delivery is ineffective for its treatment because of their dissolution and absorption in stomach and small intestine and effective concentration of drug can’t reach to the target site [70]. To overcome this problem, the dose is usually increased to ensure effective concentration at target site, however it leads towards several detrimental effects [71].

There is a need to develop a drug delivery systems that can selectively target the colorectal region. Several studies conducted on cancer have reported the experience of pain by cancer patients who are either ambulatory or receiving anticancer therapy. Thus there is also need to control the pain of cancer patients. As reported by Moertel et al., 1971 that aspirin can eradicate pain in cancer patients by providing a substantial degree of analgesia. To overcome this problem dual drug delivery of analgesic and anticancer drug is required which will provide chemotherapy as well as pain relief in cancer patients [72].

Hydrogels are three dimensional class of cross-linked polymers that can absorb large quantity of water due to their hydrophilic nature. These polymeric materials may be naturally derived, synthetic/artificial or a blend of both. Hydrogels have the property of swelling that offer moderate to high physical, chemical and mechanical stability intended for their specified use. For the fabrication of hydrogel, natural or semisynthetic polymers like alginate or carboxymethyl cellulose are widely described in the literature [73].

Carboxymethyl cellulose a biodegradable, nontoxic, inexpensive, renewable polymer is a derivative of cellulose of either bacterial or plant source. It contains carboxymethyl groups that are generated by the reaction of chloro-acetate with cellulose in alkali that produce substitutions at position C2, C3 and C6 of glucose. As a result of this substitution, carboxymethyl cellulose is hydrophilic and more responsive to the hydrolytic activity of cellulases [74]. Sodium CMC is widely used for the oral drug delivery and due to its pH sensitivity it has been potentially used for site specific drug delivery. Similarly alginates have attracted considerable attention among the commonly used polysaccharides. It has the property to protect the acid sensitive drugs from degradation by gastric juices that can achieve drug delivery in intestine. Instead of several advantages, it has the limitation of low entrapment efficiency and burst release of entrapped drug in intestine due to basic media. For this reason, several researchers have blended other polymers with alginate to overcome this issue [75].

A study conducted by Sheng et al., 2021 fabricated blend of CMC and alginate hydrogel dual loaded with methotrexate and aspirin by co-precipitation method for the treatment of colorectal cancer [69]. The hydrogel protected the anticancer drug methotrexate from being absorbed in the stomach and small intestine and successfully targeted at colorectum.

Another study conducted by Nochos et al. in 2008 used alginate with HPMC. The aim of their study was to investigate the release of model drug from alginate/HPMC gels beads. They concluded that there was significant differences in release pattern between the pure aliginate beads and alginate/HPMC beads [73].

## 13. Applications of Cellulose-Based Materials in Sustained Drug Delivery Systems

The use of natural polymers and biodegradable polymer based sustained release system for drugs were studied by formulation scientists and to achieve desired release profile. Hydrogels use in drug delivery applications was started in the 1960. Poly methacrylates-based hydrogel membranes were used in the design and study of fluoride-controlled release systems in the dental caries treatment. Surface modification is the key to enable the nanoparticle potential for its use in drug delivery. An innovative approach is the modifcation of the surface of the NPs in order to enhance its hydrophilic properties with the aid of natural cellulose-based polymer is. Covalent modification of drug NP surface would bypass or delay the phagocytosis process, thereby enhancing the drug bioavailability in the systemic circulation to reach the target. HPMC or CMC have been used in formulation of drug-based NPs due to its high biocompatibility, biodegradability, and non-toxicity. Coating of NPs with polysaccharide induces even more stability in the blood [76]. Numerouscellulose derivatives offer protective properties via their steric forces. The forces protect from having nonspecific or unbound interactions with proteins or enzymes. This in turn increases the organ or specific tissue targeting majorly due to their recognition and mucoadhesive properties [63]. Cellulose derivatives like cellulose triacetate, polycarbonate and polypropylene, can be beneficial in the formation of membrane structures with a diameters (1.5 × 10 up to 3 μm). Vinyl polymers, polyacrylates and few cellulose derivatives have been effectively used to administer transdermal route drug therapy. Cellulose or acrylic polymers are widely used in pharmaceutical coating due to their good film-forming properties that can aid both protection and sustain drug release. Cellulose and their derivatives are widely used to alter the drug release from solid dosage forms such as capsules and tablet for film formation, water retention, and to aid adhesive capabilities. The derivatives are even recognized for their use as suspending and emulsifying agent as well.

Following the failure of docetaxel (DTX) in effectively treating metastatic bone, prostate and resistant cancer (mCRPC), Hoang, Bryan, et al. (2017), have formulated 100 nm NPs of self-assembled cabazitaxel (CBZ), the clinically approved alternative to DTX, conjugated with carboxymethylcellulose-based polymer (Cellax-CBZ). The drugs achieved sustained drug release in serum at 9–10%/day. Interestingly, about 157-fold higher CBZ was delivered from Cellax-CBZ to PC3-RES in mice with prostate cancer, and found to be safe when given at a 25-fold higher dose in comparison to free CBZ. The study reported greater inhibition of tumor in different DTX-resistant mCRPC-based mice models. Luckily, Cellax-CBZ paved a path for an effective therapy of mCRPC over the already marketed CBZ [77].

Chen, Zhi, Ting Wang, and Qing Yan (2018) developed a delivery device that could entrap drug that is hydrophobic and that are pH responsive. Ibuprofen (IBU) was entrapped into β-cyclodextrin polymer (β-CDP). The core shell was crossed linked with Sodium alginate (SA), sodium carboxymethylcellulose (CMC) and HEC through physical means and capsule enhanced solubility of IBU and sustained the release at certain pH. This polysaccharide hydrogel capsule delivery system can be beneficial in preparation of oral drugs delivery [78].

## 14. Utilization of Bacterial Cellulose/Cellulose Derivative to Improve Oral/Dental Drug Delivery

Cellulose is an inexpensive, biodegradable and renewable bio-polymer mostly derived from wood, cotton and other plant materials which are rich in lignin, pectin and hemicellulose. Various researchers tried to utilize bacterial cellulose (BC) in pharmaceutical industries e.g., Badhash et al. used BC for preparation of nano-composites and bio-functionalized polymers in drug delivery [79].

BC has certain limitations e.g., solubility and instability however these limitations can be overcome with the phenomenon of regeneration. For solubility N-methyl-morpholine-oxide (NMMO) can be used for optimum solubility. NMMO treated BC is called regenerated bacterial cellulose (R-BC). R-BC has been widely implemented in drug delivery e.g it has been used for H2-blochers drugs (famotidine) and muscle relaxant (tizanidine) [80].

Researchers are of the view that BC can be used in dentistry because naturally occurring polymers like cellulose is being explored in dentistry due to its properties similar to native tissue of oral cavity. It is suggested that, in particular, there is an emerging and attractive interest in bacterial cellulose to its use as dental material [81].

Large amplitude oscillatory rheology of silica and cellulose nanocrystals filled natural rubber compounds.

Practically natural rubber (NR) is usually protected by the addition of traditional nano-particles like silica. Cellulose nano-crystal (CNC) based on cellulose biopolymer shows prospective use to partially replace such fillers for reinforcing NR nano-composites [82]. Alteration in CNC is usually carried for improving the compatibility between CNC and NR [83].

Rheological assessment of NR compounds before vulcanization is significant to know the viscoelastic characteristics for designing and processing the final product [3]. There is no agreement in the scientific community and recently it is discussed that, the chains in the matrix undertaking amplified microscopic deformation plays a essential role on the reinforcement, degeneracy and nonlinear behaviors amplitude oscillatory shear (LAOS) deformation are sensitive to filler dispersion and filler-polymer interaction, which is applied for guidance of the rubber compounding [84].

## 15. Application of Cellulose Derivatives in Oral Peptide and Protein-Based Drug Delivery

The development of an oral dosage form that provides adequate bioavailability of therapeutic peptides or proteins would certainly transform the treatment of certain diseases. As a result, numerous attempts have been made to date to achieve this objective. One of these attempts is to incorporate cellulose derivates in the preparation of the oral formulations. The primary reason for adding this kind of additives is to provide protection against gastrointestinal conditions such as acidity and the presence of proteases enzymes [85]. Additionally, it provides formulation with bioadhesive and mucoadhesive properties. Currently, the majority of bioadhesive polymers available are polyacrylic acid or cellulose derivatives [86].

Table 3 shows for example, the base layer of the gastrointestinal mucoadhesive patch system (GI-MAPS), which is intended for the oral delivery of insulin, is constructed of an ethyl cellulose polymer that is insoluble in water and protects the protein payload from hydrolytic degradation [86]. Cross-linking chitosan nanoparticles with hydroxypropyl methylcellulose phthalate, a pH-sensitive polymer, is another example in the same context. This developed insulin formulation from chitosan nanoparticles demonstrated substantial biological activity and stability in an acidic environment. The data from quantitative measurements of fluorescence and confocal microscopy analysis revealed that fluorescently labeled chitosan-hydroxypropyl methylcellulose phthalate nanoparticles increased intestinal mucoadhesion and penetration by a factor of 2–4 when compared to chitosan nanoparticles. Moreover, in comparison to oral insulin solution or insulin loaded into plain chitosan nanoparticles, the modified formulation with hydroxypropyl methylcellulose phthalate demonstrated an approximately tenfold increase in hypoglycemic effect [87]. Cellulose polymers are also used as additives in the development of pulsatile drug delivery systems (PDDS).The system is designed to deliver insulin and made up of many coating layers, one of which is an inner swellable hydroxypropyl methyl cellulose layer that improves the stability of the formulation against the proteolytic degradation [88].

## 16. Application of Cellulose Derivatives in Oral and Dental Treatment

Both bacterial and plant cellulose have great potential for application in the biomedical area, including dentistry [93]. A research group used the cellulose derivatives carboxymethyl cellulose sodium and hydroxyethyl cellulose, combined with gelatin, to get porous matrices loaded with metronidazole and given topically into the periodontal pocket [94]. Various semi-synthetic cellulose derivatives, i.e., ethylcellulose, hydroxyethylcellulose, hydroxypropyl methylcellulose, and carboxymethyl cellulose sodium salt with polyvinylpyrrolidone K30 or K90 and triethyl citrate as a plasticizer were used by Laffleur et al. [95] for designing mucosal films for the targeted delivery of allantoin to the mucosa of patients suffering from dry mouth. In the study performed by Ammar et al. [96], films composed of HPMC- or EC-based mucoadhesive films with the addition of CMC sodium, chitosan, or Carbopol were formulated and served as carriers for fluticasone propionate (2%) in mangement of lichen planus-like or other erosive lesions located on the oral mucosa.

A three-layer HPC adhesion film containing dibucaine (0.25 mg/cm^2^) was designed and used to treat oral ulcers caused by chemotherapy or radiotherapy as reported by Yamamura et al. [97]. Additionally, radiation-induced acute oral mucositis was treated by a mucosal HPC film containing tetracaine, in combination with ofloxacin, miconazole nitrate, guaiazulene, and triacetin as a plasticizer as mentioned by Oguchi and coworkers [98]. A study by Kohda et al. [99], reported that polymeric films with 30% lidocaine content prepared on the basis of EC and HPC in a 1:1 ratio revealed boosted adhesion to the buccal mucosa for about 60–120 min in clinical evaluation in all volunteers that were subjected to the clinical trials. Mohammed et al. [100] performed an investigation in which, mucosal tablets composed of hydroxypropyl methylcellulose, carboxymethyl cellulose sodium salt, Carbopol 934P, and sodium alginate and contained 20 mg of miconazole. The tablets stayed on oral mucosa for 2.45 to 3.65 h after application and released the drug in a prolonged manner compared to the commercial miconazole oral gel.

Eschel et al. [101] utilized different polymers, i.e., HPMC, Carbopol 974P, or polycarbophil, to produce hydrocortisone acetate loaded mucoadhesive tablets for managing oral mucosal lesions. Another bioadhesive tablets were developed by Kamel et al. using a mixture of HPC and carbomer, such tablets adhered to the gingiva, and provided a prolonged effect of the citrus oil incorporated in them, which was used in treating aphthae [102]. Mucoadhesive gels based on cellulose derivatives—CMC, HPC, and HPMC was used as carriers for chlorhexidine, which is a frequently used antiseptic in the treatment of gingivitis and periodontitis as reported Fini et al. [103]. Likewise, Bansal et al. analyzed the dissolution testing of CMCNa-based mucosal gel satranidazole used for the treatment of periodontitis. The results showed that the release of the drug from the carrier was favorably prolonged and controlled for 8 h, all according to Fick’s diffusion law [104].

## 17. Using of 3D-Printed Cellulose for Oral Applications

Pharmaceuticals products that can be potentially tailored to individual patients via 3D printing have received an enormous growth in recent years and are being studied more thoroughly [105]. Even though traditional pharmaceutical manufacturing processes (e.g., encapsulation, powder compaction and granulation) have a large record of use and are cost-effective due to mass-oriented production, they can be inefficient, sometimes, for the production of complex, dose-adjustable and tunable timed-release formulations [106]. It is clear that unique patient variables, such as gender, heredity, age, body mass, illness conditions, and metabolic rate, might influence dosage needs. Therefore, to overcome the constraints of conventional manufacturing processes and highlight patient focused healthcare, tailored drug therapy based on the profile of patients individually is crucial. Furthermore, medical advancements and the use of new fabrication technologies, like three-dimensional printing, have made it possible to produce more accurate and personalized medications, resulting in improved therapeutic outcomes [107].

3D printing is a manufacturing process that creates 3D objects by fusing or depositing materials into the desired shape. Various materials can be used to create 3D objects such as polymers, metal, liquids and living cells [108,109,110,111]. 3D printing, also known as rapid prototyping (RP), additive manufacturing (AM), can print items by depositing material in a layer-over-layer pattern through various printing methods such as fused deposition modelling (FDM), stereolithography (SLA), selective laser sintering (SLS), extrusion-based and inkjet [112]. By using computer-aided design (CAD), 3D printing techniques can achieve flexibility, time-efficiency, and exceptional manufacturing capabilities for manufacturing of pharmaceutical products [113].

Cellulose has been used extensively as an excipient for various types of drug products, such as extended or controlled release formulations, amorphous solid dispersions, osmotic drug delivery and bio-adhesive and mucoadhesive products. Moreover, cellulose based filaments (free or with drug) have been used to create in-house feedstock materials for fused deposition modelling (FDM) 3D printing [114]. Cellulose derivatives such as Hydroxypropyl methylcellulose (HPMC), Hydroxypropyl cellulose (HPC) and Ethyl cellulose (EC) have been popular in the recent years as 3D-printed filaments for oral application. Table 4 Shows examples of 3D-printed cellulose derivatives for oral applications purposes.

HPMC has been extensively investigated for the fabrication of oral solid formulations utilizing various inkjet, extrusion and laser 3D printing technologies. HPMC is one of the commonly used hydrophilic carriers for controlled drug delivery systems, due to its high swellability, that can significantly controls the release kinetics of an incorporated drug [115]. HPMC is characterized by high melting viscosity, low degradation temperature, a broad range of glass transition temperature (Tg) (160–210 °C) which makes it challenging to use for hot-melt extrusion (HME) processing [116]. A number of reports have shown that the addition of plasticizers such triacetin, poly(ethylene glycol) (PEG) and triethyl citrate (TEC) may be used to make HPMC filaments more easily extruded for 3D printing [117,118]. On the other hand, few reports emphasized on the importance of the concentration of the plasticizer added which have to be carefully considered as it can change the gastrointestinal motility, gastrointestinal transit time and release profile for the drug [119,120,121]. Moreover, Ahmed et al. (2021), have fabricated 3D-Printed tablets by incorporated with Self-Nanoemulsified Formulations. Tablets were formulated of a paste consisting of self-nanoemulsifying drug delivery system (SNEDDS), HPMC 4000 cp, lactose anhydrous, Polyethylene glycol (PEG) 400, Tween 80, Polyvinyl pyrrolidone (PVP). HPMC was used mainly as a gelling agent and to control the release of drugs. Authors concluded that optimizing the concentration of HPMC is essential to obtain suitable viscosity of the paste before extrusion [122].

Another example of cellulose derivatives is Hydroxypropyl cellulose (HPC). HPC is used as a binding, thickening, film coating and emulsifying agent in pharmaceutical formulations. HPC has suitable thermoplastic properties that makes it blendable for extrusion processes [107]. HPC has two glass transition temperature; First Tg is seen roughly at 4.5 °C, while the second Tg is shown above 100 °C. This is attributed to molecular mobility complexities in the polymer structure [123]. Depending on the molecular weight of HPC, the low Tg of HPC lead to low melt viscosity and fast melt flow properties. For instance, Arafat et al. (2018), have used computer aided design approach to create an immediate release tablets with unique built-in holes hot matrix extrusion and fused deposition modelling 3D printing techniques. Filaments made of HPC polymer (SSL-grade) and theophylline were employed to print capsule-like devices with interconnected blocks. The unique structure of the printed device and the use of the HPC polymer enhanced rapid drug release from the devices (>80% in half an hour) [124]. Moreover, Melocchi et al. have fabricated a capsule-shaped device for the pulsatile release orally of acetaminophen. The 3D-printed capsular device created from net HPC filaments showed a standard pulsatile–release profile, with a lag phase of around 1 h, after that complete drug release was finished within 10 min. Hence, the findings were in-line with those form capsule shells prepared using injection molding (IM) technology with the same composition [125].

## 18. Conclusions

Cellulose derivatives are remarkably being a part of dosage forms in order to satisfy specific functions or to modify the drug delivery since many drug discoveries exhibit hostile physicochemical and pharmacological properties. Cellulose and their derivatives majorly from plant source have excelled for their wider pharmaceutical uses such as additives or excipients used in nanoparticulate formulations for sustained or prolonged or controlled drug delivery. Cellulose derivatives have exhibited outstanding potential as drug carrier components in conventional, as well as matrix-based controlled release formulations such as nanoparticles, microparticles, cyclodextrins, beaded particles and even hydrogels (cross-linked). Cellulose when processed either physical or chemical modification may yield derivatives with superior properties such as biocompatible, strong, reproducible, economically cheap, even refurbished for various pharmaceutical and other industrial applications. Biopolymer derivatives like cellulose are more commonly employed to alter the release of active ingredient from solid dosage forms like capsule, tablet and even in formulations aspects like tablet binding, thickening and rheology, emulsifying, suspending, water retention and adhesive strength. Ethyl cellulose can entrap hydrophobic drugs whereas HEC and HPC can entrap large hydrophilic drugs. Both bacterial and plant cellulose have great potential for application in the biomedical area, including dentistry, in 3D printable dosage forms, and in protein and peptide delivery.

## 19. Future Prospective

Cellulose films/membranes are being used to prepare “smart” materials either by modifications through physical or chemical treatments. External parameters such as pH and temperature have been adopted as external stimuli to redesign “smart” materials for multidisciplinary applications. Stimuli-responsive based cellulose derivatives have been greatly studied over a decade, yet further research needs to be carried out because of the excellent properties and their bioapplications. Although excellent advantages of cellulose have been outlined, there is still a need of greater efforts to rediscover more practical applications in not just pharmaceutical but also biomedical fields.

## Figures and Tables

**Table 1:** Illustration of physiochemical properties of different cellulose derivatives.

Name	Ethyl Cellulose (EC)	Hydroxyl Propyl Cellulose (HPC)	Methyl Cellulose (MC)	Hydroxyl Propyl Methyl Cellulose (HPMC)	Carboxy Methyl Cellulose (CMC)	Sodium Carboxy Methyl Cellulose (Na-CMC)
Chemical Structure	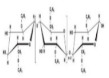	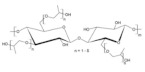	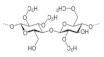	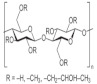	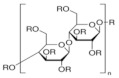	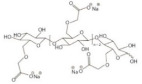
Molecular weight	454.513 g/mol	average Mn ~10,000 g/mol	454.513 g/mol	1261.45 g/mol	180.15588 g/mol	262.19 g/mol
Viscosity	5% in toluene/ethanol 80:20(lit.) is 46 cP.	The value of [η] decreases strongly with increasing temperature	2% of solution in water at 25 °C is 4500 cP.	2% of solution in water at 25 °C is 2500–5600 cP.	2 % of solution in water at 25 °C is 400–800 cP.	1 % of solution in water at 25 °C is 1500–3000 cP.
Solubility	Practically insoluble in water (room temperature)	Soluble in water, methyl alcohol, ethyl alcohol (Room temperature)	Soluble in water (Room temperature)	Soluble in water 50 mg/mL (Room temperature)	Soluble in water 20 mg/mL (Room temperature)	Soluble in water 10 mg/mL (Room temperature)
Use	Ethyl cellulose is used as a food additive and stabilizer for all animal feeds (Bampidis et al., 2020) and as an emulsifier [30]	It is used as an excipient, and topical ophthalmic protectant, solubility enhancer used for nanosuspensions, amorphous solid dispersions and poorly soluble drugs, and lubricant. Mai et al., 2020; Martin-Pastor, 2021, [31]	Bulk forming laxatives, artificial tears products [32]	Excipient in high speed tableting and capsule formulations. Controlled release agent. [33]	Used as viscosity modifiers; Emulsion stabilizer of injections; Adhesion and film-forming agents of tablets [34]	Used as a thickening agent Dispersion, emulsification, suspension, protective colloid [35]

**Table 2:** Various characterization conducted for BC or RBC based drug delivery systems.

S. No	Parameters/Characterizations	Intention of Analysis
1	FTIR	To determine the effects of various ingredients (like BC or RBC) on the purity of model drug
2	PXRD	To determine the crystallinity of BC or RBC based drugs
3	SEM	To evaluate the surface morphology of BC or RBC based drug delivery systems
4	DSC	To evaluate the interaction between BC or RBC and model drug
5	TGA	To analyze the thermo-stability of BC or RBC based drug delivery system
6	Loading efficiency	To check the percent drug loading of matrices/composites prepared BC or RBC
7	Release study	To check the release of model drug from BC or RBC based matrices or composites.
8	Release kinetics	To evaluate the mechanism of release like zero order, 1st order or pseudo order, fickian and non fickian behavior
9	Thickness	Drug matrices prepared with BC or RBC are subjected for thickness evaluation using vernier caliper.
10	Friability	To determine the withstanding power of prepared matrices based on BC or RBC.

**Table 3:** Examples of cellulose polymers used in the oral delivery of peptides and proteins.

Cellulose Polymer Used	Oral Delivery System	Peptide or Protein Used	Reference
Ethyl cellulose ^a^, Hydroxypropyl methylcellulose phthalate ^a^	Gastrointestinal mucoadhesive patch system (GI-MAPS)	G-CSF	[89]
Hydroxypropyl methyl cellulose ^a,b^	Pulsatile drug delivery systems (PDDS)	Insulin	[88]
Hydroxypropyl methylcellulose phthalate ^a^	Chitosan-based polymeric nanoparticles	Insulin	[87]
Sodiumcarboxy methylcellulose ^b^	Polymer-inhibitor conjugates	Insulin	[89]
Cellulose acetate ^a^	Gastro-intestinal patch system (GI-PS)	Erythropoietin	[90]
Sodiumcarboxy methylcellulose ^b^	Pepstatin-matrix conjugate	Pepstatin A	[91]
Carboxymethyl cellulose ^b^	Crosslinked alginate–carboxymethyl cellulose beads	Albumin	[92]

^a^ providing protection against acidic conditions. ^b^ providing mucoadhesive properties.

**Table 4:** Examples of cellulose derivatives used by 3D printing for oral applications.

Dosage Form	Cellulose Type	Application	3D Printing Technique	Reference
3D printed Tablets	HPMC	To optimize viscosity and to control the release	Extrusion printing	[122]
3D printed Tablets	HPC	to accelerate tablet disintegration and drug release.	Fused deposition modelling (FDM) 3D printing	[124]
3D-printed swellable/erodible capsular device	HPC	To create erodible capsular device for pulsatile oral application	Fused deposition modelling (FDM) 3D printing	[125]
immediate release (IR) 3D-printed oral dosage forms	HPC	To develop formulations industrially relevant	Fused deposition modelling (FDM) 3D printing	[126]
Oral tablets	EC	To develop adjustable dissolution behavior based on selective laser sintering technique	Selective laser sintering (SLS) 3D printing	[127]
Oral tablets	cellulose nanocrystals (CNCs)	Used as support materials for printing	droplet-based freeform 3D printing	[128]
Oral tablets	HPMC, HPC, EC	To investigate the effect of cellulose filaments on in-vitro drug release performance	Hot melt extrusion (HME) 3D printing	[129]

HPMC: Hydroxypropyl methylcellulose; HPC: Hydroxypropyl cellulose; EC: Ethyl cellulose; FDM: Fused deposition modelling; SLS: Selective laser sintering; CNC: Cellulose nanocrystals; HME: Hot melt extrusion.

## Data Availability

Not Applicable.
